# Supplementary material for: Extracellular Prostaglandins E1 and E2 and Inflammatory Cytokines Are Regulated by the Senescence Program in Potentially Premalignant Oral Keratinocytes
Source: Cancers (Basel). 2022 May 26;14(11):2636. doi: 10.3390/cancers14112636 (PMC9179502; doi:10.3390/cancers14112636)
Supplement: Supplementary file 1 [file cancers-14-02636-s001.zip › cancers-1665617-supplementary.pdf]

Supplementary Methods

Cell Lines used in the study

The characteristics, culture methods and properties of the LR- and HRPPOL line used in the study have been published recently and in previous publications [6, 9-10, 14]. Oral keratinocytes from the floor of the mouth were immortalized by the group of James Rheinwald. Oral keratinocytes floor of mouth line 6 (OKF6) was immortalized by the ectopic expression of the telomerase catalytic subunit *TERT* (OKF6/TERT-1*)* hitherto referred to as OKF6 [13]. Oral keratinocytes floor of mouth line 64 (OKF4/CDK4R/P53DD/TERT), hitherto referred to as OKF4, was immortalized by the defined genetic elements Cdk4 mutant, the dominant-negative p53DD peptide and ectopic expression of *TERT* OKF4/CDK4R/P53DD/TERT [17].

Conditioned medium collection

To achieve confluence in 72 hours the cells were disaggregated, the trypsin/EDTA neutralized, counted on a haemocytometer pelleted by centrifugation at 300g and the amount of protein in each cell pellet was measured and the results expressed in pg/ml medium/mg cell protein. The cell densities plated to achieve confluence in 72 hours are as follow s: OKF4, 1.1. x 10^5/square cm ; OKF6, 1 3.28 x 10^5; D19, 1.44 x 10^5; D35, 1.6 x 10^5; DOK, 7.2 x 10^4 NHOK810, 4 x 10^4; D6, 5.5 x 10^4; D9, 1.06 x 10^5; E4, 3.2 x 10^4; D25, 8 x 10^4; D4, 5.5 x 10^3; D20, 8.3 x 10^5; D34, 6.67 x 10^4 and D17, 1.33 x 10^5 .

Knockdown of p53, COX-1 and COX-2 in OKF6

The target sequences of the siRNAs are detailed in Supplementary Table S1.

Supplementary Table S1 si RNA target sequences

**siRNA**	**Target sequences**
ON-TARGETplus SMARTpool Human TP53 siRNA	5′-GAAATTTGCGTGTGGAGTA-3′<br><br>5′-GTGCAGCTGTGGGTTGATT-3′<br><br>5′-GCAGTCAGATCCTAGCGTC-3′<br><br>5′-GGAGAATATTTCACCCTTC-3′
ON-TARGETplus SMARTpool Human PTGS1 siRNA	5′-CCACAUUUAUGGAGACAAU-3′<br><br>5′-GAAUCCCUGUUGUUACUAU-3′<br><br>5′-CGAGGUGGGCUUUAACAUU-3′

	5′-UCAAGGGUCUCCUAGGGAA-3′
ON-TARGETplus SMARTpool Human PTGS2 siRNA	5′-GGACUUAUGGGUAAUGUUA-3′<br><br>5′-GAUAAUUGAUGGAGAGAUG-3′<br><br>5′-GUGAAACUCUGGCUAGACA-3′<br><br>5′-CGAAAUGCAAUUAUGAGUU-3′
ON-TARGETplus SMARTpool Human CDKN2A siRNA	5′-GATCATCAGTCACCGAAGG-3′<br><br>5′-AAACACCGCTTCTGCCTTT-3′<br><br>5′-TAACGTAGATATATGCCTT-3′<br><br>5′-CAGAACCAAAGCTCAAATA-3′
ON-TARGETplus Non-targeting pool	5′-TGGTTTACATGTCGACTAA-3′<br><br>5′-TGGTTTACATGTTGTGTGA-3′<br><br>5′-TGGTTTACATGTTTTCTGA-3′<br><br>5′-TGGTTTACATGTTTTCCTA-3′

Several rounds of optimisation were done to determine the optimised cell density, final TP53 siRNA concentrations, volume of lipid complex (DharmaFECT transfection reagent) and transfection incubation time. TP53 was optimised with cell density $2.5x10^5$ in each well of a six well plate, 100nM final siRNA concentration, and 5µl of DharmaFECT transfection reagent and 24-hours transfection incubation time. The cells were plated in antibiotic-free complete medium (DMEM with 10% FetalClone® II foetal bovine serum, 2mM L-glutamine and 0.4µg/ml hydrocortisone). The cells were incubated at 37^oC overnight. The transfection medium was replaced with complete medium containing antibiotics after 24 hours to reduce cytotoxicity. The cells were incubated for an additional 24 hours before being harvested for western blot analysis and quantitation of the level of knock down as well as conditioned medium collection.

Western blotting

Supplementary Table S2 Details of the antibodies and optimised dilutions.

**Antibody**	**Clonality**	**Species**	**Optimised dilution**	**Sources**
Anti-p16INK4a (ab81278)	Monoclonal	Rabbit	1:2000	Abcam, Cambridge, UK
P16-INK4A (10883-1-AP)	Polyclonal	Rabbit	1:5000	Proteintech, Manchester, UK
Anti-p53 (ab1101)	Monoclonal	Mouse	1:1000	Abcam, Cambridge, UK
Phospho-p53 (Ser15) (#9286)	Monoclonal	Mouse	1:500	Cell Signaling Technology, Beverly, USA
Anti-MCM7 [EP1974Y] (ab52489)	Monoclonal	Rabbit	1:10000	Abcam, Cambridge, UK
Anti-SIRT1 [E104] (ab32441)	Monoclonal	Rabbit	1:20000	Abcam, Cambridge, UK
Anti-Chk2 [EPR4325] (ab109413)	Monoclonal	Rabbit	1:50000	Abcam, Cambridge, UK
Anti-Chk2 (phosphor T68) [Y171] (ab32148)	Monoclonal	Rabbit	1:1000	Abcam, Cambridge, UK
Anti-ATM [Y170] (ab32420)	Monoclonal	Rabbit	1:10000	Abcam, Cambridge, UK
Anti-ATM (phosphor S1981) [EP1890Y] (ab81292)	Monoclonal	Rabbit	1:50000	Abcam, Cambridge, UK
EP1 Receptor (101740)	Polyclonal	Rabbit	1:200	Cayman Chemical, Michigan, USA
Anti-Prostaglandin E Receptor EP1 (ab183073)	Polyclonal	Rabbit	1:450	Abcam, Cambridge, UK

EP2 Receptor (101750)	Polyclonal	Rabbit	1:500	Cayman Chemical, Michigan, USA
Anti-Prostaglandin E Receptor EP2 [EPR8030(B)] (ab167171)	Monoclonal	Rabbit	1:1000	Abcam, Cambridge, UK
Anti-PTGER3 (ab117998)	Polyclonal	Rabbit	1:2000	Abcam, Cambridge, UK
Anti-Prostaglandin E Receptor EP4 (ab45295)	Polyclonal	Rabbit	1:1000	Abcam, Cambridge, UK
Anti-COX1 / Cyclooxygenase 1 [EPR5866] (ab109025)	Monoclonal	Rabbit	1:1000	Abcam, Cambridge, UK
Anti-COX2 / Cyclooxygenase 2 [EPR8588] (ab151571)	Monoclonal	Rabbit	1:1000	Abcam, Cambridge, UK
Anti-Notch 1 (ab87982)	Monoclonal	Mouse	1:500	Abcam, Cambridge, UK
Cleaved Notch1 (Val1744) (D3B8) (#4147)	Monoclonal	Rabbit	1:1000	Cell Signaling Technology, Beverly, USA
Anti-β-actin (ab8227)	Polyclonal	Rabbit	1:20000	Abcam, Cambridge, UK
GAPDH (14C10) (#2118)	Monoclonal	Rabbit	1:4000	Cell Signaling Technology, Beverly, USA
Anti-CREB (phosphor S133) [E113] (ab32096)	Monoclonal	Rabbit	1:5000	Abcam, Cambridge, UK
CD44 (156-3C11) (#3570)	Monoclonal	Mouse	1:1000	Cell Signaling Technology, Beverly, USA
Anti-Integrin alpha 6 [EPR18124] (ab181551)	Monoclonal	Rabbit	1:2000	Abcam, Cambridge, UK
Anti-FADS1 [EPR6898] (ab126706)	Monoclonal	Rabbit	1:1000	Abcam, Cambridge, UK

Supplementary Table S2: The dilution used for each antibody was optimised to obtain clear band on the positive controls. Wherever possible monoclonal antibodies were used to reduce unspecific binding of the protein of interest.

Supplementary Table S3 Details of the antibodies and the positive and negative controls.

**Antibody**	**Positive Control**	**Negative Control**
Anti-p16INK4a (ab81278)	HeLa	BICR3p16^{INK4A}-/-<br><br>P14^{ARF}/p15^{INK4B} +
P16-INK4A (10883-1-AP)	HeLa	BICR3p16^{INK4A}-/-<br><br>P14^{ARF}/p15^{INK4B} +
Anti-p53 (ab1101)	BICR3 Mis-sense mutation codon 282 /HEK293	BICR6 Stop codon 192
Phospho-p53 (Ser15) (#9286)	Fibroblasts irradiated with 10Gy Gamma rays and cultured for8 hours	BICR6 Stop codon 192
Anti-MCM7 [EP1974Y] (ab52489)	Senescent fibroblasts	Growing fibroblasts
**Antibody**	**Positive Control**	**Negative Control**
Anti-SIRT1 [E104] (ab32441)	Senescent Fibroblasts	Growing/quiescent fibroblasts
Anti-Chk2 [EPR4325] (ab109413)	Fibroblasts irradiated with 10Gy Gamma rays and cultured for8 hours	
Anti-Chk2 (phosphor T68) [Y171] (ab32148)	Fibroblasts irradiated with 10Gy Gamma rays and cultured for8 hours	
Anti-ATM [Y170] (ab32420)	Fibroblasts irradiated with 10Gy Gamma rays and cultured for8 hours	ATM-deficient human keratinocytes from patient AT5BI

Anti-ATM (phosphor S1981) [EP1890Y] (ab81292)	Fibroblasts irradiated with 10Gy Gamma rays and cultured for8 hours	ATM-deficient human keratinocytes from patient AT5BI
EP1 Receptor (101740)	Previously validated	Previously validated
Anti-Prostaglandin E Receptor EP1 (ab183073)	Previously validated	Previously validated
EP2 Receptor (101750)	Previously validated	Previously validated
Anti-Prostaglandin E Receptor EP2 [EPR8030(B)] (ab167171)	Previously validated	Previously validated
Anti-PTGER3 (ab117998)	Previously validated	Previously validated
Anti-Prostaglandin E Receptor EP4 (ab45295)	Previously validated	Previously validated
Anti-COX1 / Cyclooxygenase 1 [EPR5866] (ab109025)	Mock/Scrambled siRNA transfected keratinocytes	Smartpool COX1 siRNA transfected keratinocytes
Anti-COX2 / Cyclooxygenase 2 [EPR8588] (ab151571)	Mock/Scrambled siRNA transfected keratinocytes	Smartpool COX2 siRNA transfected keratinocytes
Anti-Notch 1 (ab87982)	Previously validated	Previously validated
Cleaved Notch1 (Val1744) (D3B8) (#4147)	Previously validated	Previously validated
Anti-β-actin (ab8227)	Previously validated	Previously validated
GAPDH (14C10) (#2118)	Previously validated	Previously validated
Anti-CREB (phosphor S133) [E113] (ab32096)	Previously validated	Previously validated
CD44 (156-3C11) (#3570)	Previously validated	Previously validated
Anti-Integrin alpha 6 [EPR18124] (ab181551)	Previously validated	Previously validated

Western blotting reagents

RIPA buffer (Sigma, Dorset, UK)

cOmplete™ Mini EDTA-free Protease Inhibitor Cocktail (Roche, Burgess Hill, UK

Phosphatase inhibitor (PhosSTOP; Roche, Mannheim, Germany)

NuPAGE™ NOVEX™ SDS sodium dodecyl sulphate polyacrylamide gels. (Bis-Tris pre-cast, ThermoFisher Scientific, Dartford, UK).

Skimmed milk (Marvel, Ashford, UK).

Tris Buffer Saline and Tween 20 (TBS-T) (Sigma-Aldrich, Dorset, UK).

PIERCE™ ECL Western Blotting Substrate (ThermoFisher Scientific, Dartford, UK).

Amersham ECL Prime Western Blotting Detection Reagent (GE Healthcare, Buckinghamshire, UK).

SuperSignal® West Femto Maximum Sensitivity Substrate (Thermo Scientific, Rockford, USA).

Amersham Hyperfilm ECL (GE Healthcare, Buckinghamshire, UK).

Film developer machine (Konica Minolta, Banbury, UK).

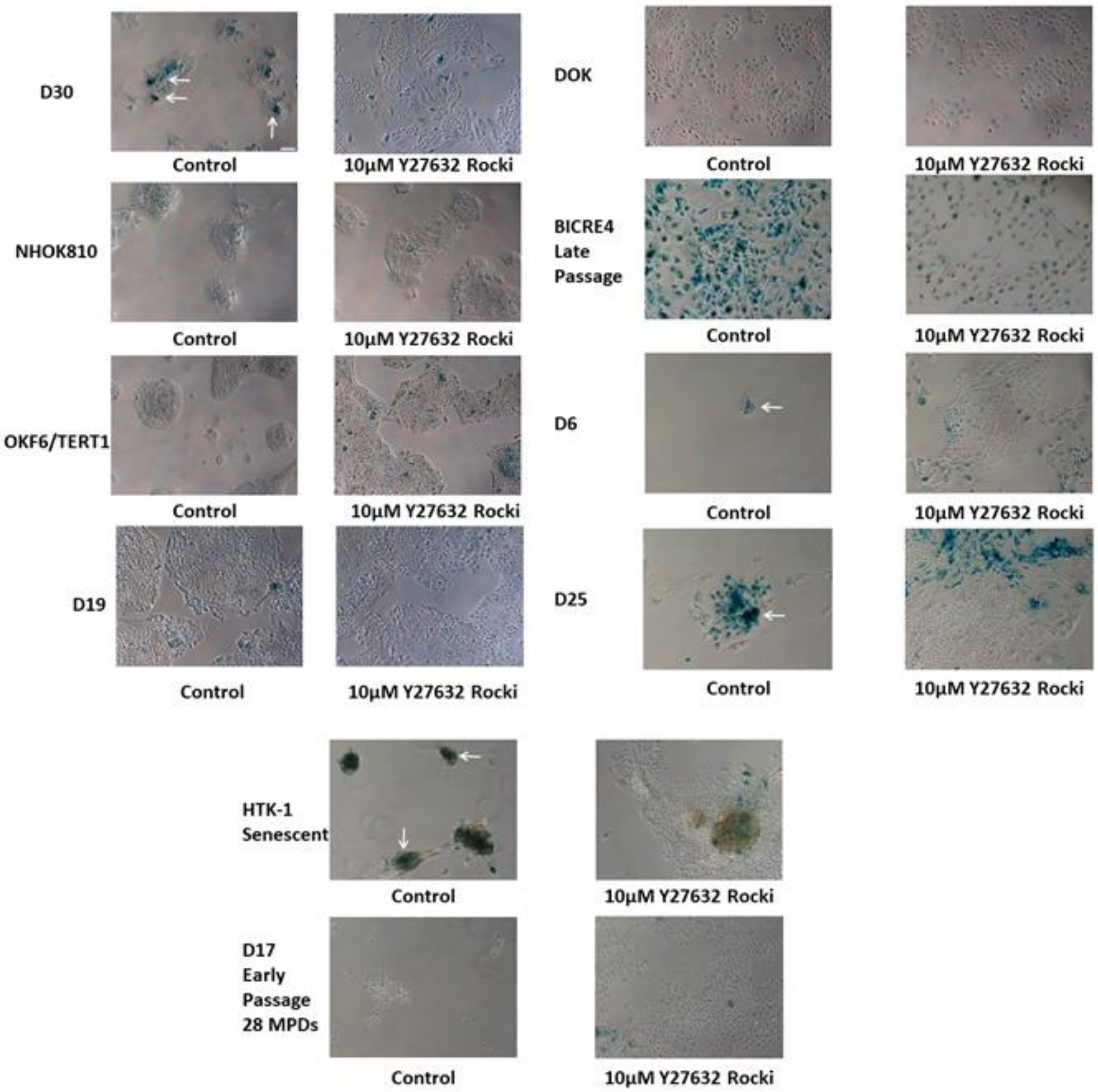

Supplementary Figure S1: SA-βGal staining of MPPOL and IPPOL keratinocytes and the effect of ROCKi.

The Figure shows the SA-βGal staining in control normal, MPPOL,

IPPOL and normal immortal OKF6 keratinocytes. Note the location of the blue cells indicated in the control and MPPOL cultures indicated by the white arrows in the centers of the colonies. These positively stained areas largely disappear following 2 weeks culture in ROCKi and the cultures continue to proliferate. The blue cells in the D25 ROCKi panel are lethally irradiated (senescent) 3T3 fibroblasts that have not been removed by the EDTA treatment. Bar = 100µm see D30 control panel).

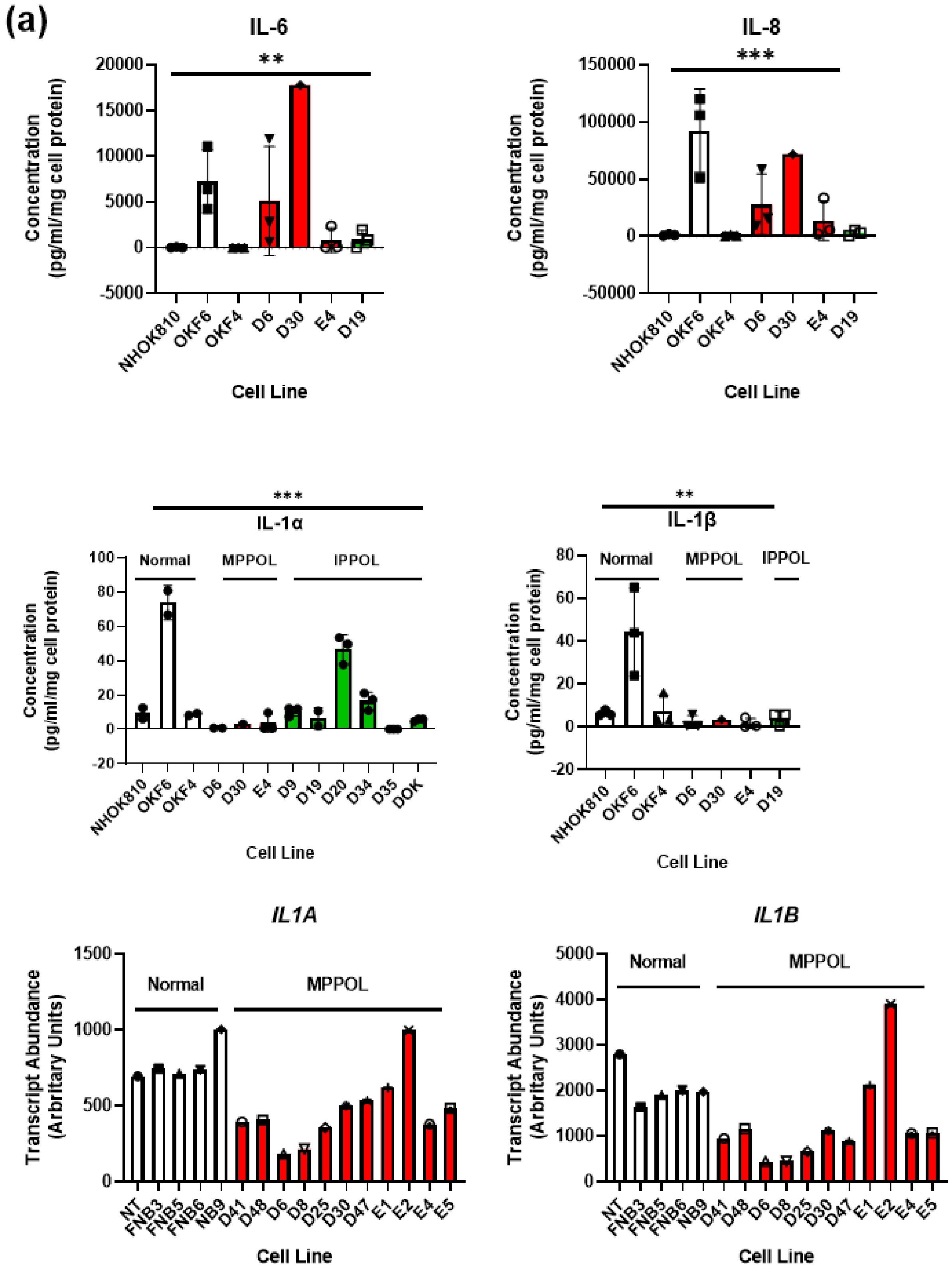
(a)
IL-6
**
Concentration (pg/ml/mg cell protein)
Cell Line
NHOK810 OKF6 OKF4 D6 D30 E4 D19
IL-8
***
Concentration (pg/ml/mg cell protein)
Cell Line
NHOK810 OKF6 OKF4 D6 D30 E4 D19
IL-1α
***
Normal
MPPOL
IPPOL
Concentration (pg/ml/mg cell protein)
Cell Line
NHOK810 OKF6 OKF4 D6 D30 E4 D9 D19 D20 D34 D35 DOK
IL-1β
**
Normal
MPPOL
IPPOL
Concentration (pg/ml/mg cell protein)
Cell Line
NHOK810 OKF6 OKF4 D6 D30 E4 D19
IL1A
Normal
MPPOL
Transcript Abundance (Arbitrary Units)
Cell Line
NT FNB3 FNB5 FNB6 NB9 D41 D48 D6 D8 D25 D30 D47 E1 E2 E4 E5
IL1B
Normal
MPPOL
Transcript Abundance (Arbitrary Units)
Cell Line
NT FNB3 FNB5 FNB6 NB9 D41 D48 D6 D8 D25 D30 D47 E1 E2 E4 E5

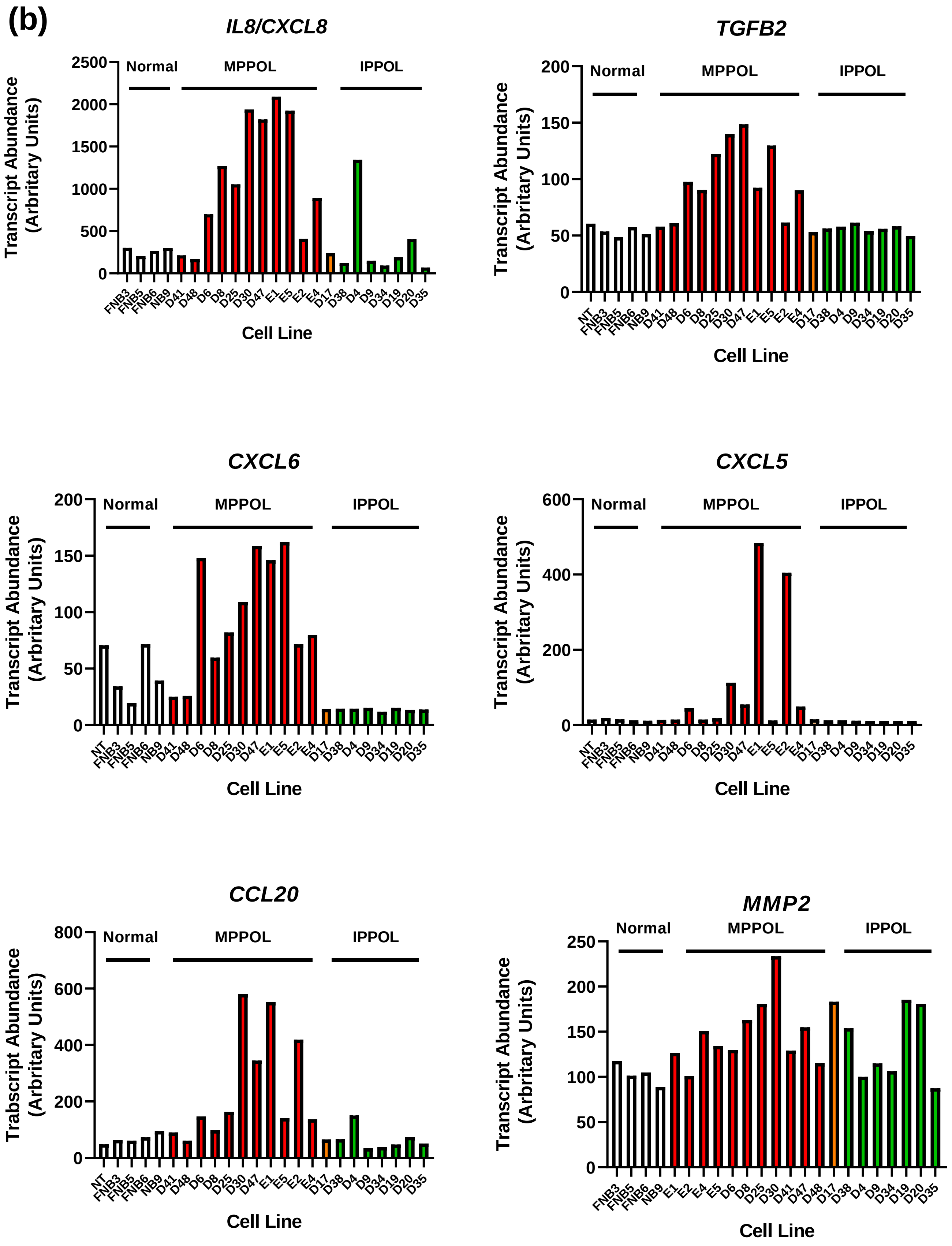
(b)
IL8/CXCL8
Transcript Abundance (Arbritary Units)
2500
2000
1500
1000
500
0
Normal
MPPOL
IPPOL
FNB3 FNB5 FNB6 NB9 D41 D48 D6 D8 D25 D30 D47 E1 E5 E2 E4 D17 D38 D4 D9 D34 D19 D20 D35
Cell Line
TGFB2
Transcript Abundance (Arbritary Units)
200
150
100
50
0
Normal
MPPOL
IPPOL
NT FNB3 FNB5 FNB6 NB9 D41 D48 D6 D8 D25 D30 D47 E1 E5 E2 E4 D17 D38 D4 D9 D34 D19 D20 D35
Cell Line
CXCL6
Transcript Abundance (Arbritary Units)
200
150
100
50
0
Normal
MPPOL
IPPOL
NT FNB3 FNB5 FNB6 NB9 D41 D48 D6 D8 D25 D30 D47 E1 E5 E2 E4 D17 D38 D4 D9 D34 D19 D20 D35
Cell Line
CXCL5
Transcript Abundance (Arbritary Units)
600
400
200
0
Normal
MPPOL
IPPOL
NT FNB3 FNB5 FNB6 NB9 D41 D48 D6 D8 D25 D30 D47 E1 E5 E2 E4 D17 D38 D4 D9 D34 D19 D20 D35
Cell Line
CCL20
Trabscript Abundance (Arbitrary Units)
800
600
400
200
0
Normal
MPPOL
IPPOL
NT FNB3 FNB5 FNB6 NB9 D41 D48 D6 D8 D25 D30 D47 E1 E5 E2 E4 D17 D38 D4 D9 D34 D19 D20 D35
Cell Line
MMP2
Transcript Abundance (Arbritary Units)
250
200
150
100
50
0
Normal
MPPOL
IPPOL
FNB3 FNB5 FNB6 NB9 E1 E2 E4 E5 D6 D8 D25 D30 D41 D47 D48 D17 D38 D4 D9 D34 D19 D20 D35
Cell Line

Supplementary Figure S2 Levels of secreted SASP cytokine protein and SASP transcript in normal, MPPOL and IPPOL keratinocytes.

(a) The top four panels show cytokine protein levels in normal NHOK810 and normal immortal OKF4 and OKF6 keratinocytes (white bars) MPPOL keratinocytes D6, D30 and E4 (red bars) and IPPOL keratinocytes (green bars). The results are significantly different by one way ANOVA ** < 0.01 *** < 0.001 (n =3) and show an increase in extracellular IL-6 and IL-8 in all 3 MPPOL lines over NHOK810 but if anything the same lines showed lower levels of IL-1α and IL-1β than normal. The bottom two panels show the levels of SASP transcript in a large panel of normal (n =5), MPPOL (n =11), D17 (MPPOL; p16^{INK4A}-/-) and IPPOL (n =7) keratinocytes mined from reference [9]. Symbols the same as the top two panels except D17 bars are orange.

(b) Transcript abundance of SASP cytokines upregulated in MPPOL keratinocytes relative to normal. Data mined from reference [9]. Symbols are the same as in (a). The data show elevated SASP transcripts in MPPOL are generally revered in IPPOL keratinocytes.

Supplementary Figure S3 ePGE regulation by DNA damage in keratinocytes.

A.

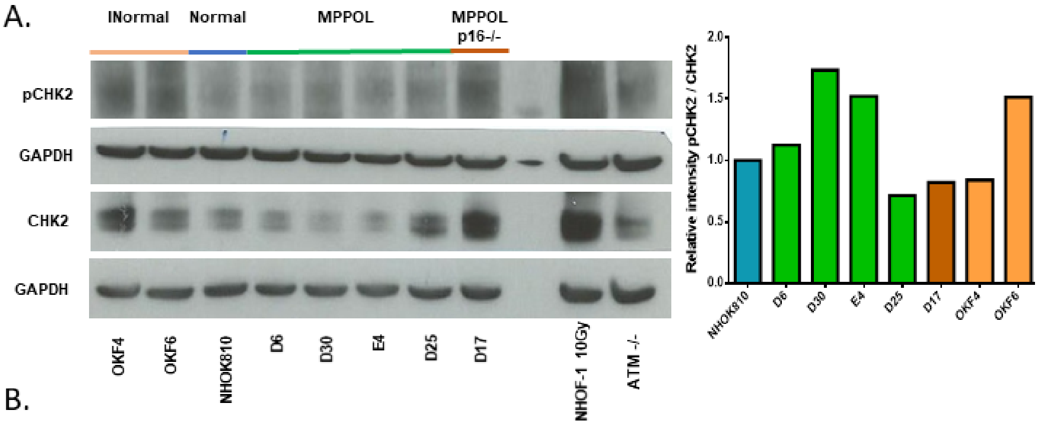

B.

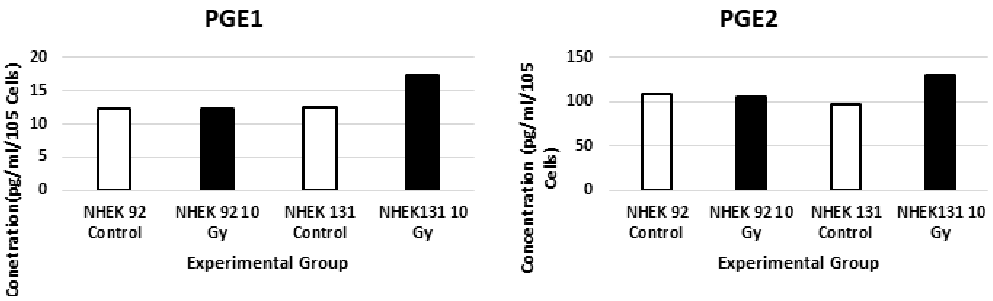

A. **CHK2 is not regulated in PPOL keratinocytes and is independent of ATM.** Western blot of (A) pCHK2 (phosphorylated at tyrosine residue T68) and total CHK2 in NHOK810, subset of the LRPPOL, OKF4, OKF6 and D17. Normal oral fibroblast irradiated with 10Gy of gamma rays (NHOF-1 10Gy) and cultured for 8 hours was used as a positive control for DDR protein phosphorylation (pCHK2 and CHK2) while normal keratinocytes from an ataxia telangiectasia patient (ATM-/-) were used as a control for ATM function. GAPDH was used as a loading control in all cases. All lanes contain equal amounts of protein (20µg), pCHK2 with histograms represent mean ratios of the protein levels relative to the total CHK2 and GAPDH loading control respectively. The figure represents data from single blot from protein extracts derived from one of the experiments in Figure 1.

B. **Measurements of prostaglandins (PGE1 and PGE2) in two commercial lines of epidermal keratinocytes (NHEK92 and NHEK131).** Both lines were irradiated at 10Gy of Gamma Rays to introduce irreparable DNA double strand breaks (IrrDSBs) and then cultured for 10 days to induce senescence. The white bars represent pre-irradiated lines while the black bars represent irradiated lines (10Gy). Concentration of the PGEs were normalised to the total cell count ($x10^5$)/ml. All data is derived from a single experiment on two lines (n=1).

A.

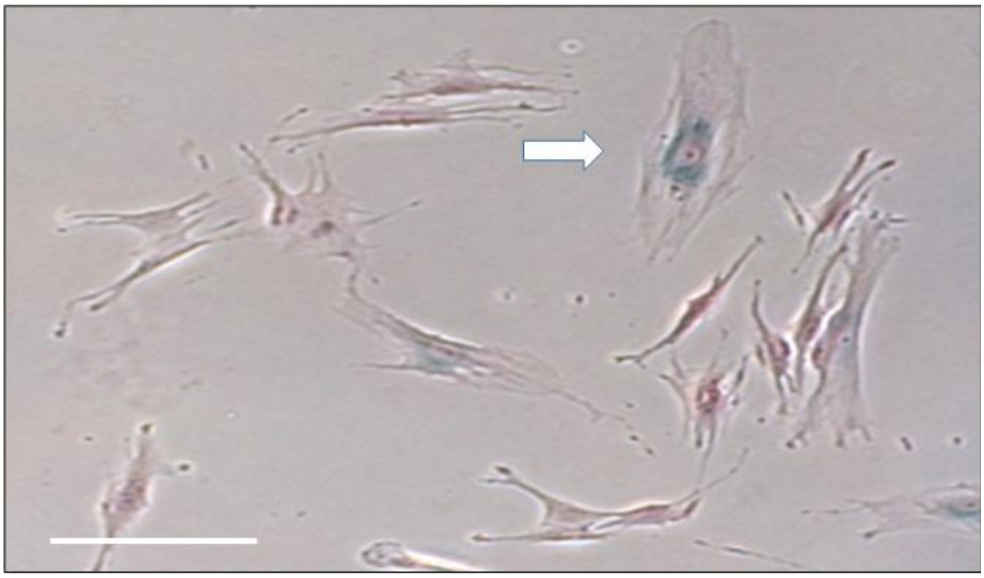

B.

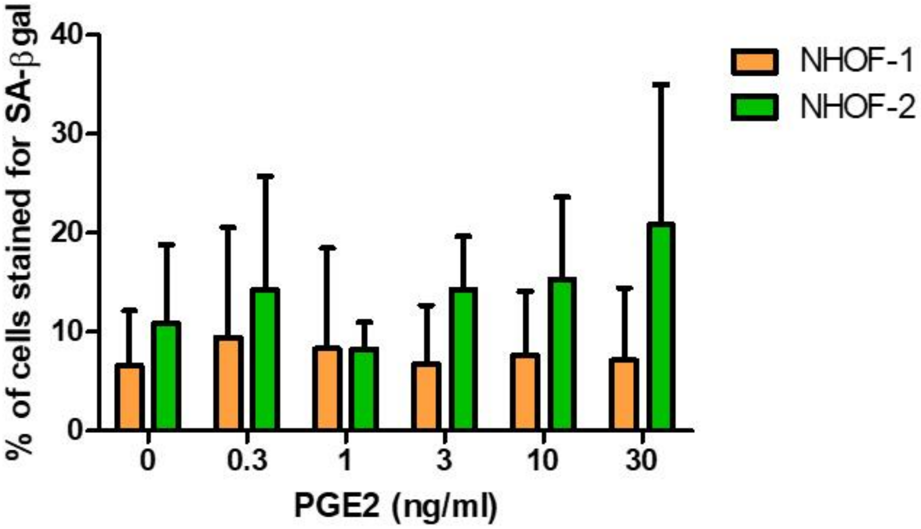

Supplementary Figure S4: Effect of PGE2 on oral fibroblasts. A. Representative image of the senescence phenotype in oral fibroblast cells stained positive blue (as shown by the arrow) for SA-β-Gal activity upon treatment with PGE2. The bar in the figure represents 100 µm. **B.** Percentage of fibroblasts positive for SA-β-Gal activity – positive blue cells. The oral fibroblasts (NHOF-1 and NHOF-2) were exposed to different concentrations (0.3, 1, 3, 10 and 30 ng/ml) of PGE2 and stained for SA-β-Gal after a 24-hour exposure. The NHOF-1 and NHOF-2 were not treated with PGE2 in the control (0 ng/ml). SA-β-Gal positive blue cells were counted in 3 different microscopic fields for each of the PGE2 concentration exposure. The histograms represent the mean $\pm$ SD of the 9 counts (3 counts x 3 repeats) from the representative results of 3 independent experiments (n=3). T-tests were done to compare means of positive stained for SA-β-Gal from each concentration in both NHOF-1 and NHOF-2 against the control (0 ng/ml). No significant difference was found between the treated and untreated groups.

Western full blots:

(A)

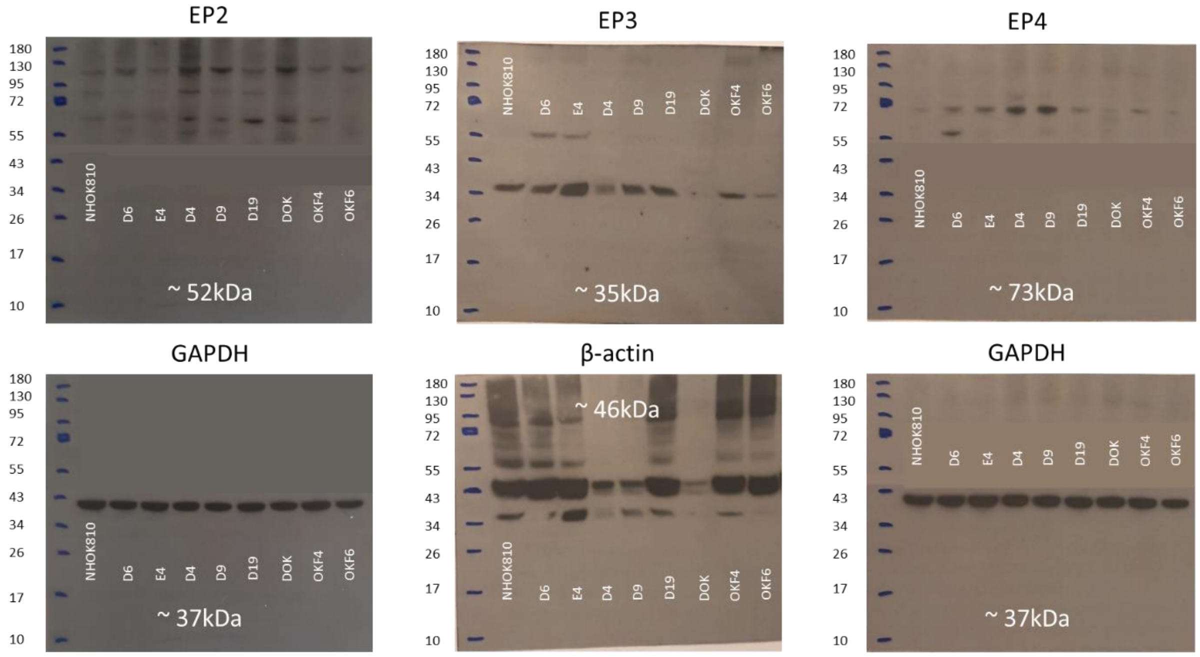

(B)

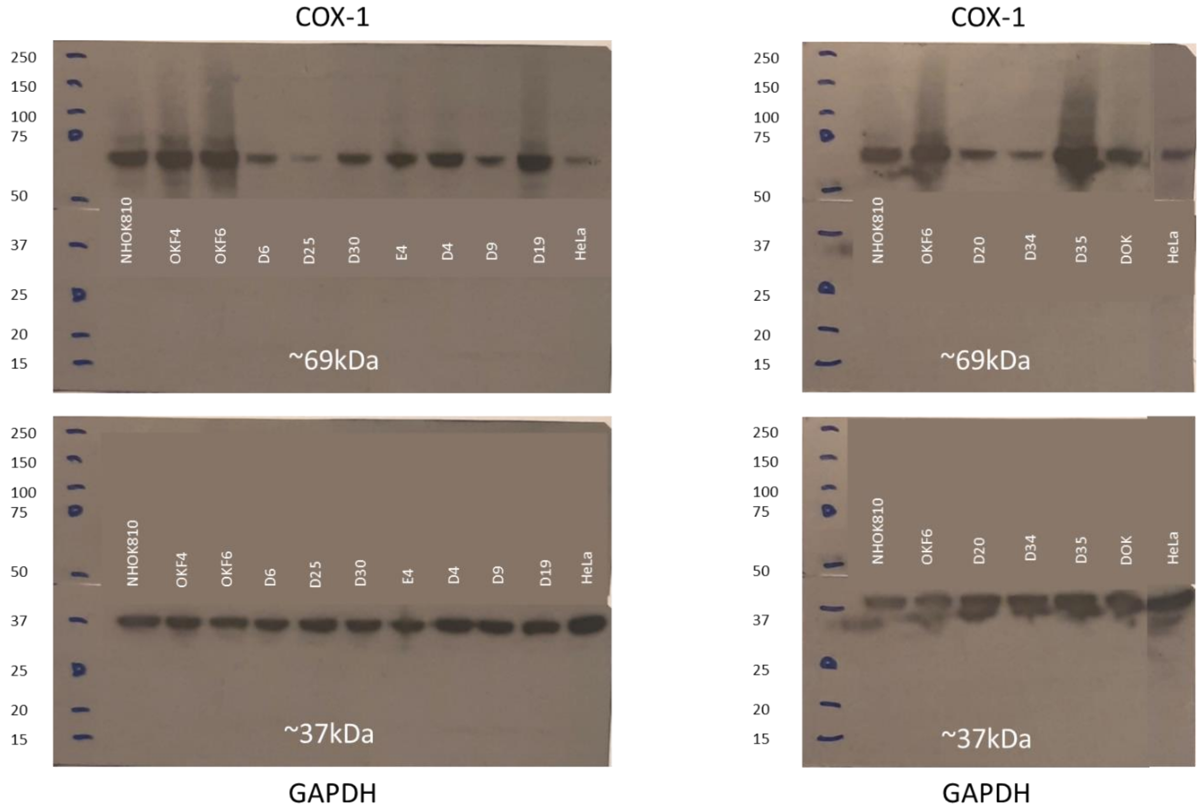

(B)

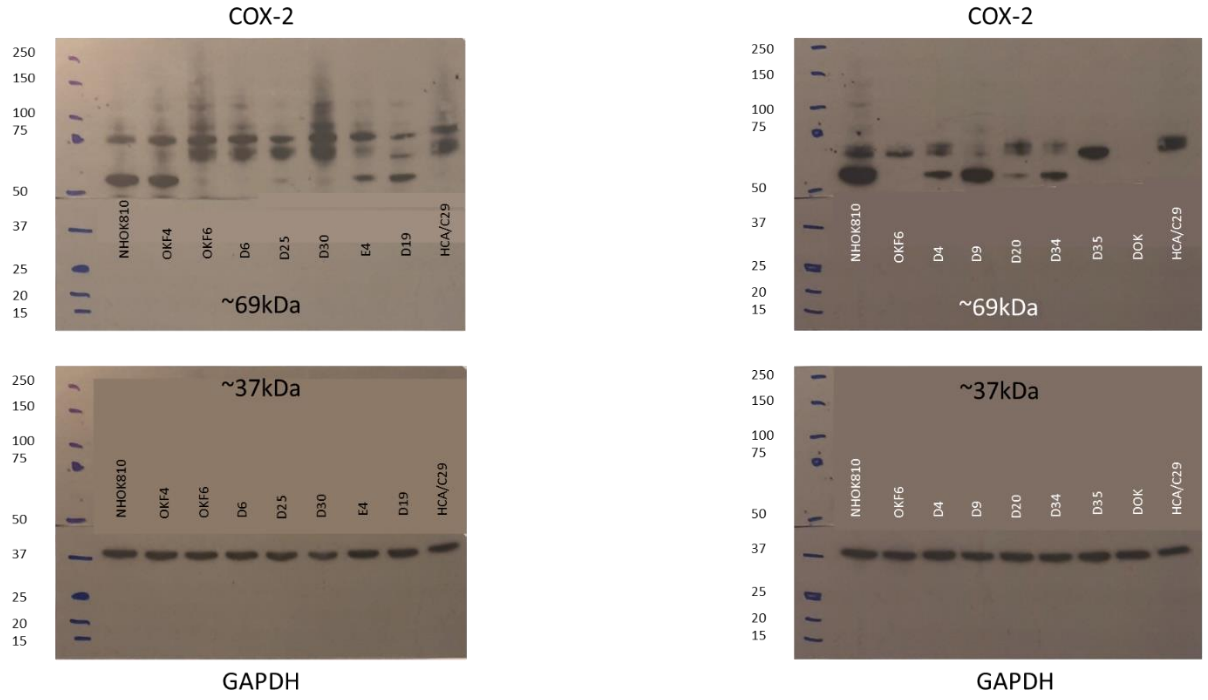
COX-2
250
150
100
75
50
37
25
20
15
NHOK810
OKF4
OKF6
D6
D25
D30
E4
D19
HCA/C29
~69kDa
COX-2
NHOK810
OKF6
D4
D9
D20
D34
D35
DOK
HCA/C29
~69kDa
~37kDa
GAPDH
~37kDa
GAPDH

(A)

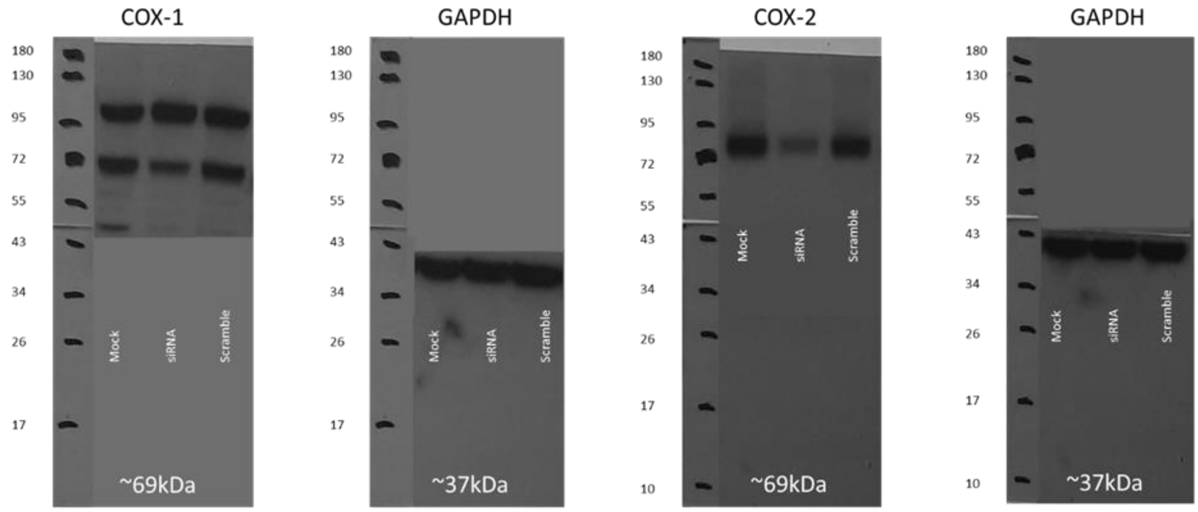
COX-1
180
130
95
72
55
43
34
26
17
Mock
siRNA
Scramble
~69kDa
GAPDH
~37kDa
COX-2
10
~69kDa
GAPDH
~37kDa

(A)

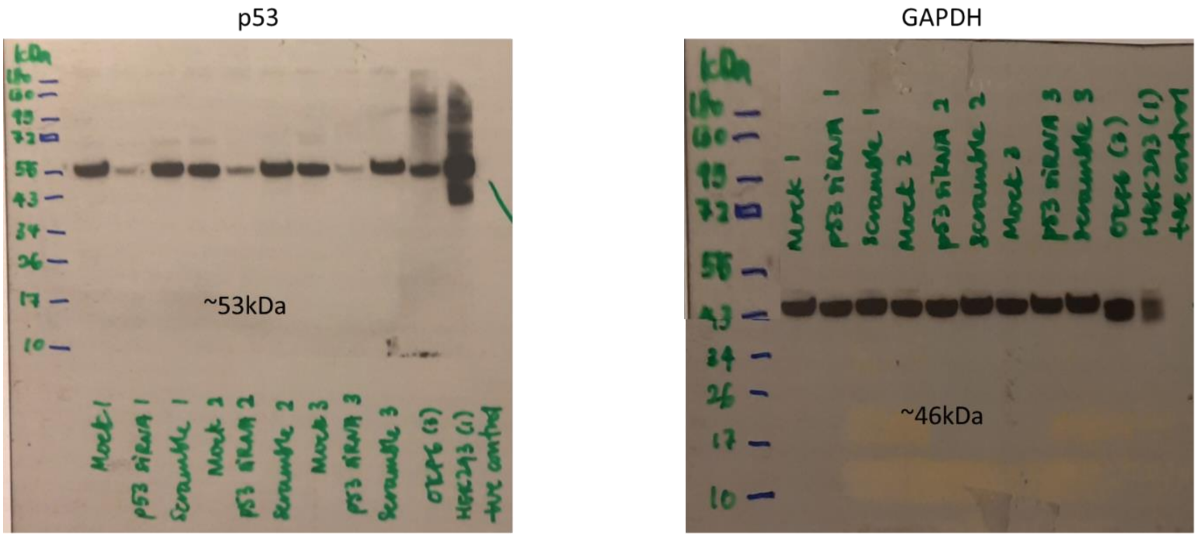
p53
kDa
~53kDa
Mock 1
p53 siRNA 1
Scramble 1
Mock 2
p53 siRNA 2
Scramble 2
Mock 3
p53 siRNA 3
Scramble 3
GAPDH
~46kDa

(B)

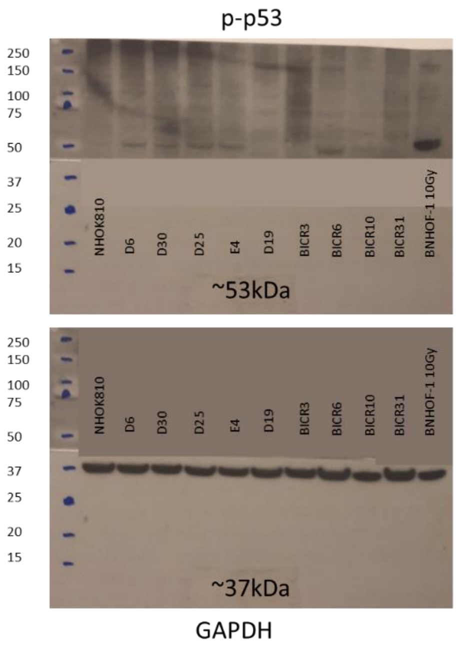
p-p53
250
150
100
75
50
37
25
20
15
NHOK810
D6
D30
D25
E4
D19
BICR3
BICR6
BICR10
BICR31
BNHOF-1 10Gy
~53kDa
~37kDa
GAPDH

(C)

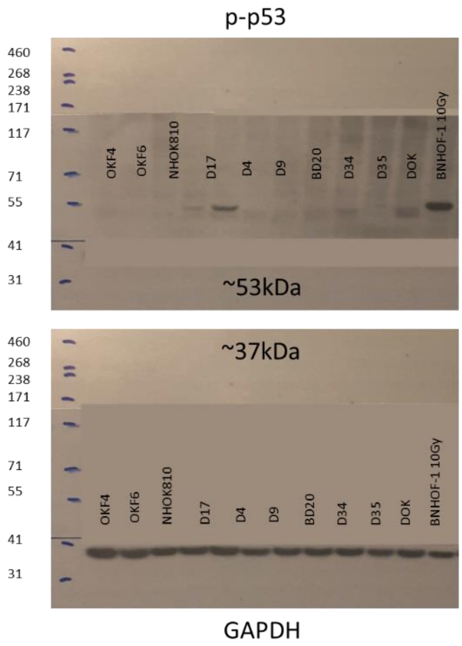
p-p53
460
268
238
171
117
71
55
41
31
OKF4
OKF6
NHOK810
D17
D4
D9
BD20
D34
D35
DOK
BNHOF-1 10Gy
~53kDa
~37kDa
GAPDH

(B)

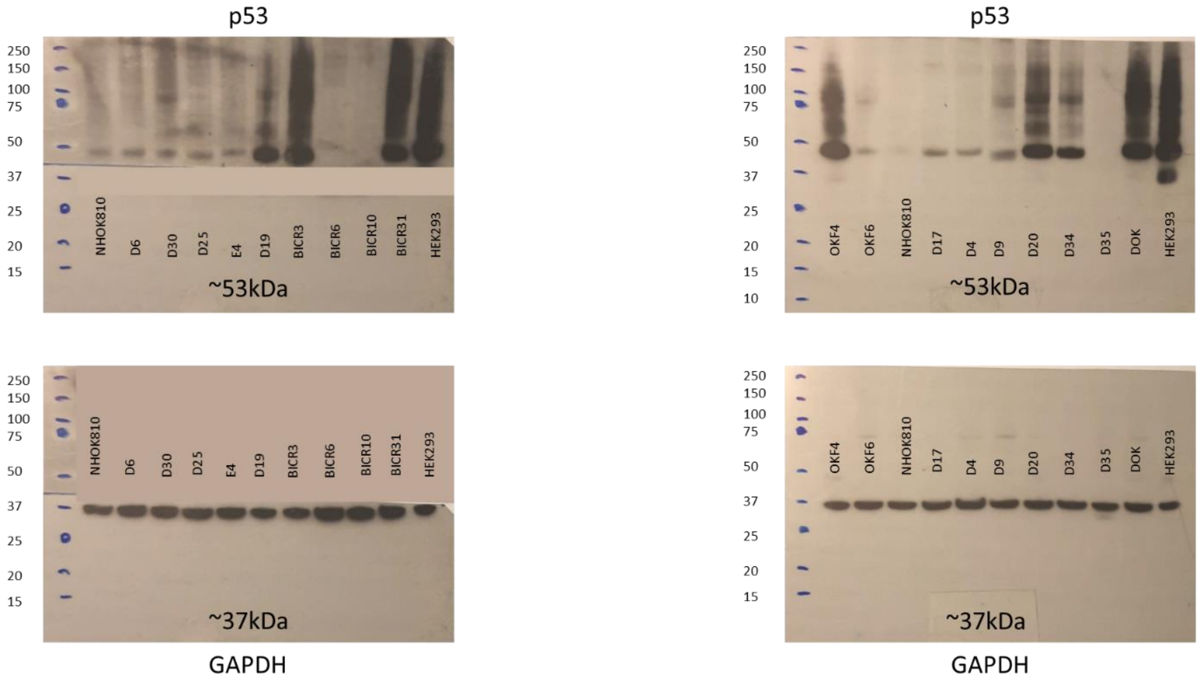
p53
250
150
100
75
50
37
25
20
15
NHOK810
D6
D30
D25
E4
D19
BICR3
BICR6
BICR10
BICR31
HEK293
~53kDa
p53
10
OKF4
OKF6
NHOK810
D17
D4
D9
D20
D34
D35
DOK
HEK293
~53kDa
~37kDa
GAPDH
~37kDa
GAPDH

(C)

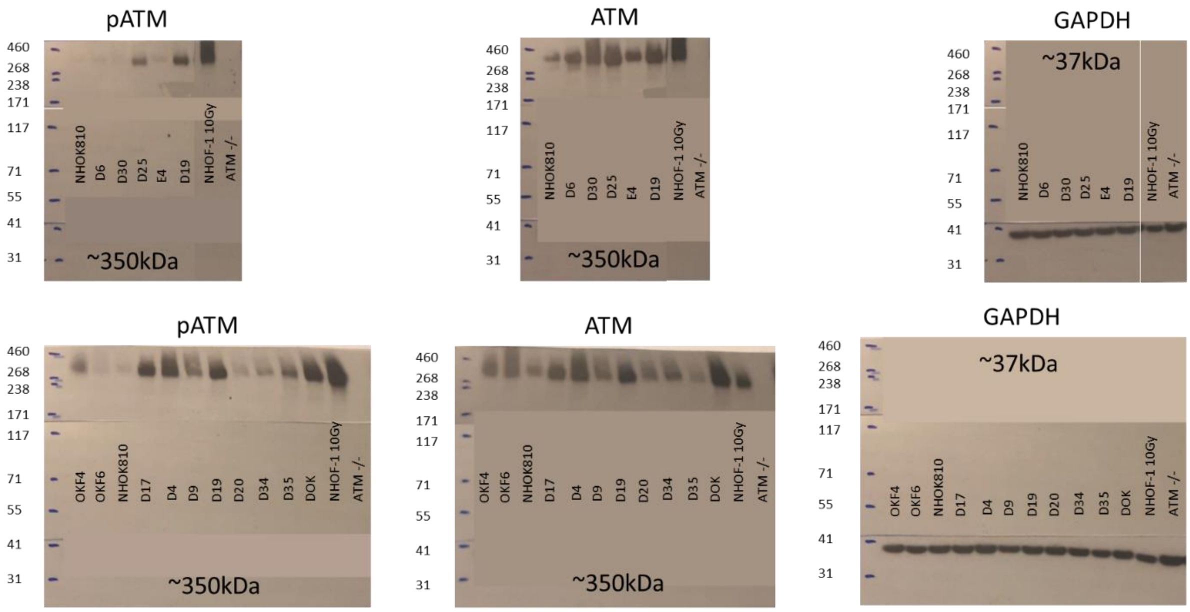
pATM
ATM
GAPDH
460
268
238
171
117
71
55
41
31
NHOK810
D6
D30
D25
E4
D19
NHOF-1 10Gy
ATM -/-
~350kDa
~350kDa
~37kDa
pATM
ATM
GAPDH
OKF4
OKF6
NHOK810
D17
D4
D9
D19
D20
D34
D35
DOK
NHOF-1 10Gy
ATM -/-
~350kDa
~350kDa
~37kDa

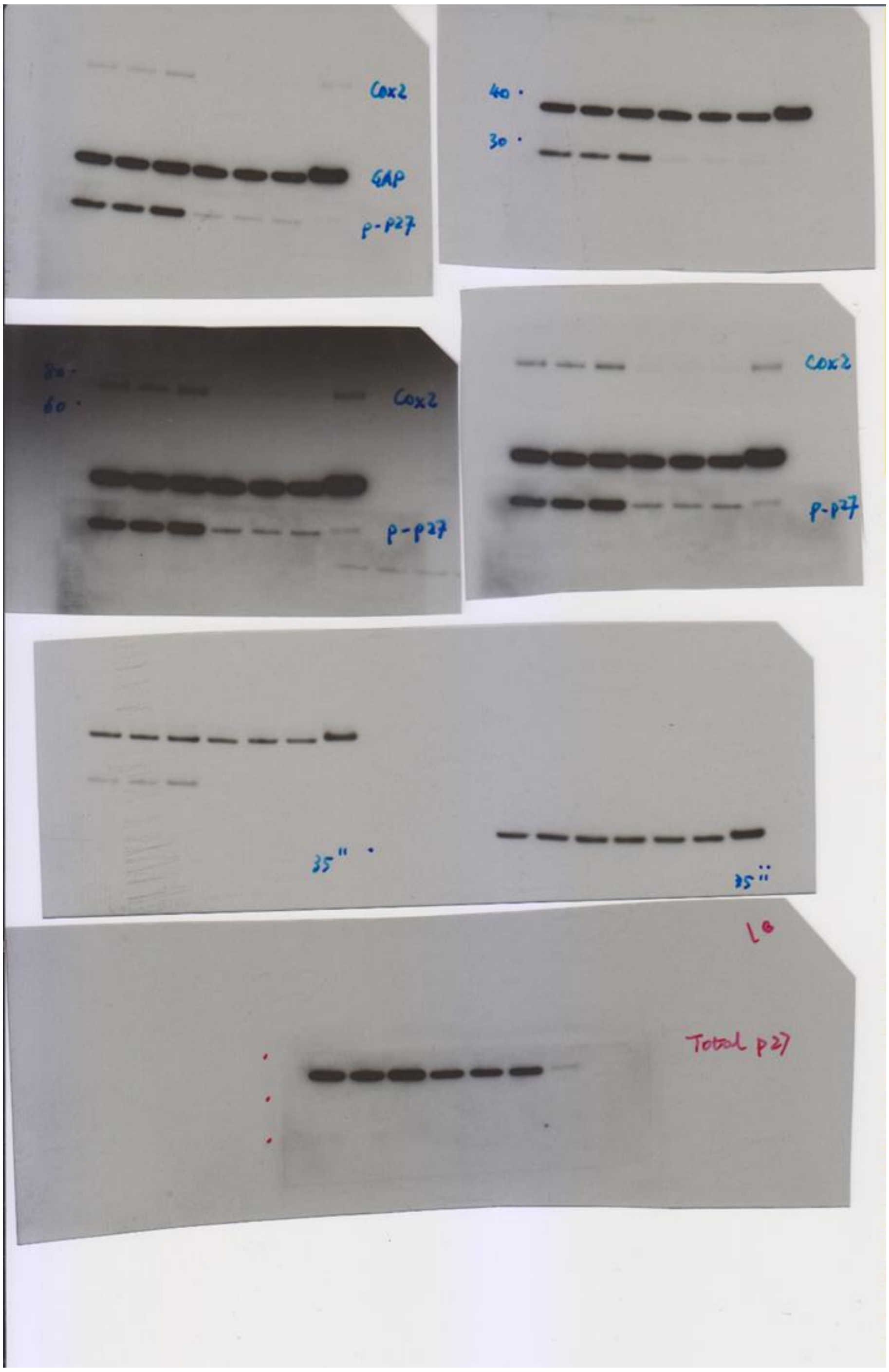

Supplementary Figure S5. The whole Western blots are shown. Ladder used for (A) was Thermo Scientific PageRuler Prestained Protein Lad-der (Cat no. 26617, ThermoScientific, Watham, USA), (B) Precision Plus Protein Standards Dual Color (Cat no. 161-0374, BioRad, Hertfordshire, UK) and (C) was HiMark Pre-Stained Protein Standard (Cat no. LC5699, ThermoScientific, Watham, USA).
